# Does Play-Based Learning Support Children’s Everyday Resiliency? A Cross-Case Analysis of Parents’ and Kindergarten Teachers’ Perceptions of Play-Based Learning as a Precedent to Young Children’s Coping During the Pandemic-Affected 2020–2021 School Year

**DOI:** 10.3390/children11111378

**Published:** 2024-11-13

**Authors:** Lisa Fyffe, Angela Lewis

**Affiliations:** 1 Department of Occupational Therapy, Colorado State University, Fort Collins, CO 80523, USA; 2 Center for Educator Preparedness, School of Education, Colorado State University, Fort Collins, CO 80523, USA; angela.lewis@colostate.edu

**Keywords:** everyday resiliency, play-based early childhood education, kindergarten transition

## Abstract

Background: Play has long been credited with fostering self-regulation in young children, though few studies have examined how children draw upon early childhood experiences with play to navigate adversity later in childhood. The purpose of this study is to describe the facets of the children’s everyday resiliency that were attributed to their play-based experiences by parents and teachers as they reflected on the children’s kindergarten experiences during the COVID-19 pandemic. Methods: We used a cross-case study design to examine the positive coping strategies parents and teachers observed in three five-year-old girls and one six-year-old boy entering kindergarten during the 2020–2021 pandemic-affected school year. We recruited parent participants based on their child’s enrollment at a Reggio Emilia-inspired, play-based early childhood center in Northern Colorado for three or more years prior to entering kindergarten. Once parents enrolled in the study, we extended an invitation to their child’s kindergarten teacher to participate. Data included three one-hour interviews with each participant, field visits during remote learning, and artifact collection over the course of the school year. Results: Cross-case analysis revealed four themes: “Willing to Adjust”, “Understands the Situation”, “Uses Advanced Language”, and “Embraces Challenge”. Participants describe these children as willing to adjust to public health orders, having a deep understanding of the pandemic’s societal repercussions, using sophisticated language to process adversity, and embracing the challenge of becoming kindergarteners. Conclusions: Participants believed these children’s experiences with play-based learning promoted the interdisciplinary development of their social language, persistence, altruism, and cognitive flexibility; these traits fostered the children’s positive coping strategies throughout the pandemic-affected kindergarten year.

## 1. Introduction

The residual effects from the COVID-19 pandemic have placed more emphasis on addressing children’s mental health across early care and education settings. Since the onset of the pandemic in March 2020, children have experienced unprecedented levels of occupational deprivation resulting in a shadow pandemic of mental health decline, suicide, school-based learning loss, obesity, and screen addiction [1,2,3,4]. While the COVID-19 pandemic impacted the lives of all children, not all experienced the same degree of adverse health effects. Dodd et al. (2022) found that children who engaged in adventurous play during COVID-19 lockdowns showed fewer symptoms of anxiety and depression than peers who did not play. Much of the research on the pandemic’s impact on young children’s learning focuses on learning loss [5,6,7]. Few make visible young children’s sophistication in response to COVID-19 health orders. The findings in this study describe how play promoted the interdisciplinary development of children’s mental health and well-being, cognitive flexibility, and language development, thus contributing to their everyday resiliency during the pandemic.

Playful contexts tend to generate scenarios of manageable stress and cognitive dissonance, wherein children begin to develop positive coping mechanisms that prepare them for everyday stress and conflict [8,9]. For example, as children experience manageable stress during playful contexts (i.e., low-stake dysregulation) the ability of self-regulation is gradually refined [10]. Children who constructively respond to everyday life challenges experience higher positive effects and fewer internalizing symptoms of anxiety and depression [10,11]. For young children, experiencing play is critical in the development of their emotional well-being and positive mental health [12,13].

Vygotsky [14,15] was the first to postulate that play promoted children’s positive mental health by fostering self-regulation, an intrinsic process of behavioral, emotional, and cognitive modulation in response to environmental demands. Vygotsky believed children developed self-regulation during play through a combination of self-talk and self-reflection [14,15,16]. Self-talk occurs when children internally narrate and process social interactions during play, therefore language acts as a protective mechanism against adversity and stress. Self-reflection occurs when children migrate between fantasy and reality to construct and enact social roles during play; thus, play prompts reflective thinking and social awareness.

The novelty encountered by children during play encourages their exploration, negotiation, discovery, and solving of problems [16] and these playful experiences teach children to view change and challenge as positive forms of stress that can be mastered through ingenuity [8,9,10]. Because play often involves creative and fluid circumstances, children experience recurring dysregulation as they navigate an array of dynamic roles and experiences in the context of imagination. Solutions to these naturally occurring dilemmas require agency, reflection, and intention on the part of the child; thus, concurrently demanding and facilitating children’s focus and flexibility [15]. Therefore, children develop self-regulation through play as they manage their impulses, align their behavior with group play norms, and devise novel solutions to challenge in response to social demands and peer feedback [8,15,16,17].

As children encounter low-stakes dysregulation early in life, they practice altering their emotions and behavior in response to social cues. Over time, children systematically build upon these foundational schemas as they learn to manage more complex dysregulation in response to increasingly complicated scenarios [18]. Self-regulation fosters children’s positive engagement with teachers and participation in learning, allowing children to manage their emotions, follow directions, build supportive relationships, and pursue goal-directed activities [13,19,20,21]. Children who develop a flexible adaptation of self-regulation skills throughout early childhood are more likely to understand and apply these skills in novel situations with familiar adults and peers, leading to successful experiences in kindergarten and later lifelong success [22,23].

In early care and education centers, children may be exposed to play-based learning, a pedagogical approach to develop young children’s social emotional competence and promote prosocial brain development. Adult guidance and specific information are only provided to extend children’s learning beyond what they achieve on their own [24,25]. Because play-based learning promotes children’s autonomy, agency, and engagement, children experiencing play-based learning in early childhood develop robust communication, social relationships and flexible approaches to learning [4].

Play has a clear and positive influence on children’s mental health and well-being, most notably in the areas of emotional regulation, cognitive flexibility, and social reciprocity, all key predictors of a successful transition into kindergarten [19]. Play is transformative for young children, yet little research exists that studies how children draw upon skills developed in their prior playful experiences to navigate future life transitions and everyday stress over time. In this case, children drew upon the affective skills of thoughtfulness and attunement to the feelings of others, the cognitive skills of demonstrating positive approaches to learning, perseverance and persistence, problem solving, and the use of sophisticated language, as described by parents and teachers. The purpose of this paper is to illustrate parents and kindergarten teachers’ observations of how four children drew upon their play-based learning backgrounds to navigate their kindergarten transition during the pandemic-affected 2020–2021 school year.

## 2. Materials and Methods

This manuscript is part of a larger, longitudinal study of how four children fared in kindergarten following play-based education at a Reggio Emilia-inspired early childhood center. The data describing the everyday adaptability of these children is presented in this manuscript. We used Yin’s [26] cross-case study approach to design and implement this study. Data collection occurred from September 2020 to May 2021. This study was approved by the Colorado State University Institutional Review Board (approval 19-9519H) and the participating school district’s Research Director.

### 2.1. Participants

Using Yin’s [26] purposive sampling technique, we recruited parents of children enrolled at an early childhood education center (subsequently referred to as “the center”) with plans to register their child for kindergarten for the 2020–2021 school year. The center is a laboratory school associated with a four-year university, primarily tuition based, and integrates the Reggio Emilia-inspired educational philosophy with a play-based learning pedagogy. The Reggio Emilia-inspired philosophy of early childhood education emphasizes artistic expression, child-led exploration, engaging environments, and collaborative relationships to promote children’s curiosity and joyfulness with learning [27]. Play-based learning is a pedagogical approach to early childhood education grounded in guided play, where the adult curates a learning context towards an educational goal and the child maintains agency and some degree of freedom to explore and discover while learning [24]. The center and public school district is located in northern Colorado. The United States Census Bureau (2022) describes northern Colorado as 84.5% white with a median household income of USD 72,932 and with 56.6% of adults having earned a bachelor’s degree or higher [28].

Parents of children who had been continuously enrolled at the center for at least three consecutive years were recruited via an email invitation from the center’s Executive Director. Once a parent agreed to participate in the study, they provided contact information for their child’s kindergarten teacher, who was then emailed an invitation to join the study. We recruited all participants in the summer preceding the children’s kindergarten enrollment.

Yin [26] describes four participant cases as ideal for cross-case study research; four cases are large enough to offer multiple participant vantage points on a shared phenomenon yet small enough that the research team can capture in-depth and nuanced perspectives. Therefore, we enrolled four participant clusters as cases; see Table 1 for details (pseudonyms used throughout). A participant cluster ideally included a parent and a kindergarten teacher both providing perspectives on a child’s kindergarten experience. Mothers were the parent informants in all four participant clusters; all were married and employed at the time of this study. Mothers were the only parents who volunteered for the study, though all parents were invited. Three of the four kindergarten teachers participated as informants; one teacher declined participation due to concerns regarding additional responsibility during the pandemic-affected school year. Kindergarten teachers represent elementary schools with three different curricular foci: Core Knowledge; International Baccalaureate; and Science, Technology, Engineering, and Math (STEM). The kindergarten classrooms were not play based.

Participants reported a range of perspectives and prior experiences related to play-based learning prior to joining this study. Two parents reported enrolling their child at the recruitment site because they preferred play-based learning while two others said play-based learning was not a factor in their enrollment decision. One teacher taught at a play-based preschool prior to becoming a kindergarten teacher while the remaining two teachers had no direct experience with play-based learning.

### 2.2. Data Collection

Each parent and teacher participant completed three in-depth semi-structured interviews lasting approximately one hour at the start, midpoint, and conclusion of the school year; see Table 2 for sample interview questions. The focus of each interview was driven by the aim of the study, which was to understand how children drew upon their play-based backgrounds to navigate their kindergarten transition and the stress of daily life during the pandemic. Interview protocols were developed and revised with input from the research team. Once the team agreed on a set of questions, the protocol was trialed with a current kindergarten teacher and a parent of a child transitioning into kindergarten from play-based learning who was not enrolled in the study. Based on their feedback, the protocol was finalized. We recorded all interviews, which each lasted approximately one hour and were conducted via video conferencing, and a professional service transcribed them verbatim.

We used separate but parallel interview protocols for the parent and teacher groups; this capitalized on each participant’s unique vantage point, while also allowing for comparisons of perspectives within and across cases. In the case of Nadine, who had only a parent participant, we used the same parent interview protocol with her mother as we did with the other parent participants. We then blended Nadine’s data within the cross-case analysis as appropriate.

### 2.3. Data Analysis

Cross-case study design is unique from case study design. For example, a recent study on play in early childhood education that utilized case study design focused on an already identified phenomenon, seeking an in-depth understanding of a single case [29]. However, this study seeks to compare similarities and differences across multiple cases to arrive at shared themes and uncover novel findings. Congruent with Yin’s (2018) case-based approach to cross-case analysis, the research team completed within-case analysis prior to cross-case analysis. Each parent and teacher interview were read multiple times for familiarity, and this resulted in a preliminary list of seven-to-ten major topics specific to each case. Because we were engaged with participants over three interview cycles across a full school year, we were able to triangulate and probe participant responses by comparing teacher and parent insights by case. For example, when Isaac’s teacher described Isaac’s prowess with social language and relationship building in the classroom, we investigated further by asking Isaac’s mother how Isaac’s language had developed over time and what influences she saw as contributing to his language skills. From this inquiry, we learned that Isaac had acquired language at an early age and that Isaac’s mother believed play-based learning immersed Isaac with emotional intelligence and social reciprocity which fueled his connectedness with others.

At the conclusion of the within-case analysis, we merged the full data cache along prominent conceptual clusters to begin cross-case analysis. Adaptation was a recurring theme in the data and became an axial code linking the data across cases and interview cycles. We operationalized adaptation into a probing question (How are parents and teachers describing the children’s responses to change and challenge throughout their kindergarten transition and school year?) and constructed cross-case themes using thematic analysis based on this guiding question.

We described and defined each cross-case theme and selected representative quotes to illustrate the theme’s meaning. See Table 3 for the details of this process.

### 2.4. Internal Validity

We took many steps to safeguard the integrity of our data analysis. We remained engaged with participants for a full academic year which allowed for data triangulation and opportunities for member checking among participants. We practiced highly disciplined subjectivity through data trail audits and constant comparative analysis within and across cases [30]. We held recurring meetings to build consensus with our findings throughout the study.

### 2.5. Pandemic Influence

The original intent of this study was to provide an in-depth examination of how children fared in kindergarten following play-based learning. However, given the amplitude of children’s distress during the 2020–2021 school year with fluctuating instructional format shifts and day-to-day uncertainty, coping and resiliency became a much larger factor in influencing how children acclimated to their role as kindergartners to meet classroom expectations and advance their learning. Therefore, everyday adaptability became a prominent focus of data collection efforts as to the pandemic revealed the permeating importance of play in children’s development as learners.

## 3. Results

We identified four themes that illustrate how these children adapted to adversity and change over the course of their kindergarten year: (1) willing to adjust, (2) understands the situation, (3) uses advanced language, and (4) embraces challenge. Each theme is connected to the participant’s expression of their individual beliefs, perceptions, and observations of the child of focus through stories, encounters, and conversations occurring over the course of this study. Parent and teacher participants ascribed these traits to the children’s positive coping strategies throughout the pandemic-affected kindergarten year. Parents do not typically have the knowledge base to express the value or connection of everyday playful experiences to child development and learning [31]. Therefore, we make the deductive leap and connect the development of these traits acquired during their time at the play-based preschool these children attended. This deductive, causal reasoning is explored in the Discussion section of this study. See Table 4 for full descriptions and definitions of each theme.

### 3.1. “Willing to Adjust”

This theme centers on the parent and teachers’ observations of how the children adapted to kindergarten and responded to change and adversity. The 2020–2021 pandemic-affected school year was fraught with uncertainty for these children. They were unable to participate in orientation events and school tours due to public health orders limiting in-person events and they began their kindergarten year in remote learning, an unfamiliar educational context. These children also navigated four district-wide instructional format shifts between remote and in-person learning as well as classroom and school-specific instructional format shifts in compliance with local public health protocols.

Participants described how the children demonstrated their adaptability to the ever-changing public health orders through their attitudes and actions. Addy’s mother and teacher shared examples of her flexibility in response to the evolving school year while Isaac’s mother described his resiliency and perseverance. For example, Addy’s teacher reflected on how Addy remained engaged and productive with kindergarten during both remote and in-person learning, saying “I think she’s just taking it all in stride and rolling with it”. Addy’s mother had initially reported that Addy was unfocused during the early weeks of kindergarten’s remote learning format. However, she believed Addy’s focus improved drastically once she was able to attend in-person school and “see” what a kindergarten class should look like. Overall, Addy’s mother believed that Addy kept a positive attitude given the changing expectations and protocols for school and the need to participate in learning through a computer, saying “kindergarten changes and she changes”.

Similarly, Isaac’s mother described Isaac’s attitude about remote learning by saying: “Even through those times where he was less enthusiastic, he still was great. Even though he didn’t really enjoy being online, he did it”.

The parents also reflected on how their children were willing to alter their social and leisure activities to better align with public health restrictions on social gatherings. Leah’s mother described how Leah approached choosing between horseback riding or soccer as an extracurricular activity.

“She was really being mindful about group sizes and how we’re being exposed and is pretty insightful as to knowing this is what we need to do to stay safe. So, horseback riding was a really great activity choice, because it really only involved her and the instructor”.

(Leah, 2nd Parent Interview, p. 13)

Nadine’s mother also noted Nadine’s contentment with more solitary activities at home, a shift from pre-pandemic norms where she would have enrolled in after school activities and socialized with friends each weekend.

“I feel like everything has been so different and closer to home [since the pandemic], and we have a much tinier life. [Nadine] now loves just putzing around and taking her time exploring the yard or going for walks and I think that’s really helpful for her and she really enjoys it.”

(Nadine, 2nd Parent Interview, p. 7)

### 3.2. “Understands the Situation”

This theme illustrates how the children’s understanding of the pandemic and its related public health orders fostered their responses to navigating kindergarten in the context of COVID-19. Parents and teachers described the self-reflection and thoughtfulness the children showed in response to the pandemic’s public health orders. Their stories and observations illustrated how the children contributed to community health and appreciated the reasons for the various restrictions they had to follow. Nadine and Leah’s experiences provided the strongest examples of this theme, as both girls used their artistic creativity to bring joy to their neighbors and reinforced public health orders at home. Nadine’s mother shared:

“I feel like [Nadine]’s getting that the rules are for everyone to be safe. I think she’s in tune with that, and says things like ’since COVID, we have to do all of these things like stay away from people and wear masks”.

(Nadine, 2nd Parent Interview, p. 5)

Leah’s mother described how Leah would correct her if she inadvertently acted against public health orders:

“She’s stopped me a few times when I’ve offered a big package of pretzels to her at-home learning pod classmates. She’ll say, “No mom, they need to be individually wrapped”. So clearly, she’s been trained, and she very much understands the situation”.

(Leah, 2nd Parent Interview, p. 16)

The children also recognized more vulnerable members of their community and created opportunities to bring joy to others during this challenging time. Leah’s mother told a story where, after a week of remote learning, Leah and her at-home learning pod classmates decided to make a hopscotch course on the sidewalk for pedestrians, saying the following: “They wanted everybody coming by our house to try it, and it was just really, really fun and showed them kind of making lemonade out of a lemon situation”.

Nadine’s mother described how Nadine and her brother understood the impact of social isolation and made sidewalk chalk art for an elderly neighbor who lived alone and was not receiving visitors during the pandemic:

“We have a neighbor down the street who’s in her 80s and when she’s on her porch, the kids do chalk drawings for her. It’s just a very sweet way of showing our neighbors that we care without physically interacting so much”.

(Nadine, 2nd Parent Interview, p. 8)

### 3.3. “Uses Advanced Language”

This theme describes the protective role expressive language played in promoting the children’s adjustment to life during the pandemic. Parents and teachers illustrated how these children used expressive language to articulate the support they needed to process the challenge and uncertainty of the pandemic-affected school year. The children’s prowess with labeling their feelings and communicating their needs to trusted adults enabled them to find positive coping mechanisms to mitigate their stress reactions. Isaac’s experience provided the strongest example of this theme. Isaac’s mother believed that Isaac’s language skills gave him a sense of confidence because he knew he could articulate his feelings and obtain the support he needed to handle unsettling events:

“When he went back after break, he was so sure he was just going to do the kiss and go line, but then he said, “I’m feeling nervous. I’m not so sure about the [drop-off] line”. He wasn’t in distress, but he was able to articulate, “I guess I do want you to walk me to my classroom door, and here are the reasons why”. He knows that he can articulate what he’s feeling and that he can advocate for himself with his words”.

(Isaac, 2nd Parent Interview, p. 19)

The use of language also enabled the children to articulate their needs and advocate for themselves at home and at school. Leah’s mother described a time of frustration during the final week of remote learning before winter break:

“I think we just hit a breaking point there and she just said, “Mom, I’m so tired”. I think that she was able to see and recognize that she needed her own break, and her way of creating it was to lay down in her room”.

(Leah, 2nd Parent Interview, p. 11)

Nadine’s mother also described how Nadine coped with difficult feelings at home. She spoke of a time during the school year when Nadine was exhausted from remote learning and asking when she could do in-person school again:

“In those moments, she would go up to her room and just take some time by herself. She had some music up there that she liked, and she would draw by herself. And she has one of those squeezy stuffies and would say, “Sometimes when I’m angry I just need to squeeze my stuffy”.

(Nadine, 2nd Parent Interview, p. 18)

### 3.4. “Embraces Challenge”

This theme centers on how the children responded positively to the many forms of challenge they encountered over the course of their kindergarten year. While the pandemic brought about many unique obstacles, the children also encountered the expected stress of developing their academic skills and adjusting to kindergarten life. Parents and teachers described how the children approached these new expectations with confidence and ingenuity, traits teachers found to be exceptional during the pandemic-affected school year when so many children in their classrooms struggled with self-esteem.

Leah’s teacher described how Leah embraced her role as a student when she was asked to work through a difficult math concept by saying the following: “She doesn’t want things to feel easy all the time, because she sees the good that comes from that challenge, and she wants to do well and apply what she knows”.

Isaac’s mother also noticed how Isaac thrived in response to having more responsibilities by observing “Isaac has a little bit more purpose to his day as a kindergartener. He’s got a job to do when he goes to school, he’s challenged and he’s finding a lot of satisfaction in that”.

The children also employed creative problem solving as they encountered the academic challenges of kindergarten. Nadine’s mother described Nadine’s ingenuity with completing her 100-day project when she could not count to 100.

“So, she wrote out to 100 in groups of 10, and she didn’t know what all the numbers were called but she knew the pattern. She said, “I know that eight comes next, and then nine comes after that”. It’s really cool to see her use what she had learned even when she didn’t know everything”.

(Nadine, 2nd Parent Interview, p. 2)

## 4. Discussion

The purpose of this qualitative cross-case study was to describe how four five-year-old children drew upon their experiences with play-based learning to navigate the challenges of daily life as a kindergartener during the pandemic. Participants described these children as willing to adjust to the ever-changing school year, understanding the pandemic and its associated public health rules, using advanced language to communicate their needs, and embracing the challenge of navigating a substantial life transition during an uncertain time. Participants believed that play-based learning contributed to these children’s adaptability by equipping them with many ways of learning, an appreciation for the perspective of others, command of expressive language, and perseverance when faced with challenge.

The literature addressing how children develop everyday adaptability through exposure to manageable stress suggests that play fosters children’s positive mental health via three main pathways: First, play immerses children in novelty and imagination, which encourages children to develop many ways of thinking and a positive view of uncertainty [10,11]. Second, play fosters children’s self-regulation through a combination of self-talk and self-reflection; by developing complex social language and habits of introspection, children create positive narratives to explain conflict and ambiguity [14,15]. Finally, play encourages children to develop social awareness as they recreate and enact social scenarios and roles. This drives children to take the perspective of others, thus expanding their ability to delay gratification and temper their impulses for the common good of the group [8,16].

Entering kindergarten with well-established coping strategies enabled the stress of navigating school during the pandemic to feel manageable to these children, thus they could focus on their role as students rather than succumbing to disquietude. When the children encountered pandemic-related stress, they talked through their feelings, connected with others, and found joyfulness in everyday moments. The children responded to the expectations of kindergarten with persistence and self-assurance, which fueled their competency and growth as students. Teacher participants shared many stories of the challenges they saw in their classrooms with children feeling anxious and unsure of themselves, and therefore being unable to assimilate to classroom expectations. However, teachers also consistently reported that the children who were the focus of this study were adept at meeting classroom expectations, and this facilitated their growth and academic advancement as students. The children illustrated both competency and autonomy, which allowed them to overcome adversity and successfully acclimate to their new role as kindergarteners.

These findings align well with Vygotsky’s (1966, 1978) description of self-regulation as the merging of internal dialogue and behavioral responsiveness to social demands. Participants shared many examples of the children using their language skills to describe adverse emotions and to express their needs to trusted adults. For example, Isaac and Leah were both able to tell their parents when they felt overwhelmed by the pandemic, which enabled them to respond with empathy and understanding. The children also accepted alterations in their daily routines as public health orders evolved. They created moments of connection despite social restrictions, such as Nadine creating sidewalk chalk for her elderly neighbor. By sharing their experiences and finding joyfulness in everyday moments, the children maintained an overall positive mental state and avoided the most severe manifestations of pandemic-related duress.

Moreover, we argue that the development of these traits is connected to the play-based preschool these children attended. The current literature continues to underscore play as a pedagogical approach [16] and method of teaching to support children’s problem-solving skills, agency, and self-confidence [17,29]. In this cross-case study, the themes of “Willing to Adjust”, “Understands the Situation”, and “Embraces Challenge” highlight foundational interdisciplinary skills acquired through play in the early years that lead to later academic achievement and lifelong success [32,33]. For example, the theme of “Willing to Adjust”, highlights general positive approaches to learning, including flexibility in thinking, and a disposition towards perseverance. There is also a presence of problem-solving as demonstrated in the children’s choice-making strategies. In the theme of “Understands the Situation”, the children’s active participation in their hybrid classroom and neighborhood community points towards a sophisticated level of thoughtfulness as well as their growing expertise in applying flexible adaptation to everyday life. There is also a presence of social–emotional development specific to understanding and responding to the feelings of others. This includes understanding, explaining, and applying the rules associated with public health orders. In the theme of “Embraces Challenge”, the children demonstrated confidence, and inventiveness, and showed greater resiliency and persistence on appropriately challenging tasks. Lastly, the theme of “Uses Advanced Language”, shows the children’s ability to use language to express their thoughts and needs, and their engagement in reciprocal, extended conversations by using sophisticated language to label their feelings and advocate for their needs, often using complex syntax and grammar. These early and emergent language skills that naturally arise in playful contexts link to later literacy development, including reading and writing [34].

These findings add to the literature by illustrating how parents and teachers viewed play-based learning’s effect on the adaptability of these children navigating both major life stressors and expected life transitions. Studies of play and adaptability have focused on play’s immediate effect on children, for example, Dodd et al. (2022) study of how play affected children’s mental health during the pandemic. Other studies focus on young children not understanding how to regulate their emotions [5], learning challenges [6], and learning loss [7] during the pandemic-affected school year. Our study offers an example of how children draw upon play-based backgrounds to employ effective coping strategies in the face of a global pandemic and substantial life transition.

## 5. Conclusions

Early childhood professionals continue to grapple with a shadow pandemic of developmental regression and social isolation. Understanding factors that enabled children to mitigate the most devasting impacts of the pandemic and its related public heath orders can inform parenting practices and education policy for the next generation. Despite the existing tension between play-based learning and traditional standardized learning, the value of play is held in high regard in early childhood education and viewed as a pathway for learning and to disrupt societal inequalities [16,17,29,31,35]. Our study offers insight into the play and adaptability relationship by exploring connections observed by parents and teachers related to how children develop positive health from play-based learning.

Adaptability arises when children are emotionally regulated rather than emotionally reactive. When children have opportunities to develop positive coping mechanisms in the context of everyday stress, they are better prepared to employ and expand upon these mechanisms as they encounter increasingly more complex stress. These findings offer an in-depth description of young children’s adaptability during the pandemic-affected school year from the perspectives of their parents and teachers. Although this case study makes visible the children’s sophistication in response to the COVID-19 health mandates, the research team would also like to name a particular level of sadness in the resilience that was required of the children. Early childhood professionals and policy makers can use these findings to inform their decisions in creating policy and practices that foster children’s positive mental health. We suggest further research on the causal correlation between play-based learning and young children’s resiliency independent of the pandemic.

## 6. Limitations

Congruent with cross-case study, these findings describe the adaptability and positive response to challenge seen in four children following immersion in a play-based early childhood program. While the themes align with play-based learning scholarship, there are many influences on a child’s development over time; therefore, it is not possible to exclusively attribute these findings to play-based learning alone. For example, the children who were the focus of this study were well supported by their involved parents and compassionate teachers. They also lived in a community with many outdoor leisure offerings for young children that were accessible during the pandemic, such as horseback riding and skiing. The participants in this study did not identify as members of a historically marginalized group or share concerns with economic insecurity. Similar studies on play make visible the concept of play as an economic privilege [35]. We did not specifically ask parents about their beliefs towards play as in previous studies [31] nor their views on public health orders or how they responded to everyday stress. The teacher and parent participants believed that play-based learning was a significant positive influence on the children’s mental health and were able to make explicit connections between competencies the children developed during play and the mechanisms through which they coped with the pandemic and kindergarten transition.

## Figures and Tables

**Table 1:** Descriptors of the participant clusters (code names used).

Descriptors	Addy	Isaac	Leah	Nadine
Gender	Female	Male	Female	Female
Age (at initial interview)	5 years, 7 months	6 years, 1 month	5 years, 6 months	5 years, 6 months
Birth order	2nd of two siblings	1st of two siblings	2nd of two siblings	2nd of two siblings
Kindergarten curricular focus	Core Knowledge	Science, Technology, Engineering and Math	International Baccalaureate	International Baccalaureate

**Table 2:** Sampling of interview protocol questions by participant group.

Participant Group	Sample Question
Teacher and parent interview protocols	Describe a time when [child] encountered change or challenge during the school year. How did [child] respond?
	How do you think play-based education contributed to [child’s] kindergarten experience?
Parent interview protocol	Outside of remote learning, how would you say [child] life as a kindergartener has been different, in comparison to [sibling] experience or the experience you imagined [child] would have?
	What has [child] said about all the changes they have experienced with kindergarten over the past school year?
Teacher interview protocol	How have the public health orders related to COVID affected your students’ kindergarten transition and school year?
	Outside of remote learning, how would you say [child] kindergarten experience has been different compared with previous kindergarten cohorts you have taught?

**Table 3:** Willing to adjust theme construction: within-case analysis to cross-case analysis.

Phase 1: Axial Code “Adaptability” Identified “Adaptability” as a Significant Construct in the Data Set.	Phase 2: Probing Question How Did Participants Describe These Children’s Responses to Adversity and Challenge?	Phase 3: Data Groupings Identified the Descriptive Patterns Across the Cases.	Phase 4: Cross-Case Themes Developed Cross-Case Themes Describing Children’s Adaptability.
Sample of Data Excerpts for “Willing to Adjust”			
“There’s just a willingness to adjust to [public health orders], whereas before it was a play date with multiple friends at once. Now it’s just this idea of one other friend we can get together with”. [Leah, 2nd Parent Interview]	“She really rolled with it, which was amazing to me. She just really seemed like, “Okay, that’s fine. We’ll do this.”” [Nadine, 2nd Parent Interview]	“It was just surprising to me that being a five-year-old, she was okay getting online and she was okay talking her class that way. For the most part she’s just adapted”. [Addy, 2nd Parent Interview]	“He is just in command, from online, to hybrid, to back online, to fully in person. He’s just doing great at all of it, and we haven’t had huge rocky transition periods. We really haven’t”. [Isaac, 2nd Parent Interview]

**Table 4:** Everyday adaptability theme definitions, descriptions, and illustrations.

Theme	Definition	Description	Illustrative Quotes
“Willing to adjust”	Expressing beliefs, perceptions, or sharing observations illustrating the child’s productive adaptability to change and adversity.	Stories of how the child altered their attitude and actions to remain engaged with kindergarten.	“I think just kind of fostering that idea of let’s be flexible, let’s figure this out. I think she understands all of that. I really think she’s adapted pretty well to everything, because there’s so many new rules” (Leah, 2nd Parent Interview, p. 13)
“Understands the situation”	Expressing beliefs or sharing observations illustrating the child’s awareness of the COVID-19 pandemic and public health orders.	Stories, perceptions, or observations of how the child responded to the pandemic and its related public health orders with self-reflection and thoughtfulness.	“She’s even like, “Oh, well, I love to hug my friends, but I understand that I can’t do that”. I know adults that don’t understand that. Like, all the time”. (Nadine, 2nd Parent Interview, p. 14)
“Uses advanced language”	Expressing beliefs or sharing observations illustrating the child’s use of language to self-advocate and connect with others.	Stories, perceptions, or observations of how the child used language to self-advocate and connect with others.	“His language skills enable his ability to share how he feels on a different level than a lot of other kids. I think that he is very in tune with his emotions, and I think he’s very fair in sharing what he needs”. (Isaac, 2nd Teacher Interview, p. 8)
“Embraces challenge”	Expressing beliefs or sharing observations illustrating the child’s positive response to challenge.	Stories, perceptions, or observations of the child responding positively to challenge.	“She is right where I want her to be academically because she’s a very hard worker who takes feedback well and tries to apply what she knows to make progress with things like her reading and writing”. (Addy, 2nd Teacher Interview, p. 2)

## Data Availability

The data presented in this study are available on request from the corresponding author due to participant privacy.
